# Letter to Editor: “Virtual 3D models, augmented reality systems and virtual laparoscopic simulations in complicated pancreatic surgeries state of art, future perspectives, and challenges”

**DOI:** 10.1097/JS9.0000000000002782

**Published:** 2025-06-14

**Authors:** Jiaqi Yao, Chao Wu, Chi Ma

**Affiliations:** a Department of Anesthesiology, The First Affiliated Hospital of Dalian Medical University; b Department of General Surgery, The First Affiliated Hospital of Dalian Medical University

*Dear Editor,*

We read the article by Laga Boul-Atarass *et al* with great interest^[1]^.The paper systematically reviews the independent applications of 3D modeling, AR/VR (Augmented Reality/Virtual Reality), digital twin, and AI (Artificial Intelligence) technologies in pancreatic surgery, providing important references for the field. We highly appreciate this study, and if the following aspects can be fully considered in the article, it may promote further development in the field of pancreatic surgery.

The complexity of pancreatic surgery – rooted in its unique anatomical location, diverse pathophysiology, and technical challenges – dictates that single technologies rarely cover the entire diagnostic and therapeutic process. For example, Schlanger et al.^[2]^ pointed out that the existing AI research mostly focuses on a single link (such as complication prediction) and lacks a full-process system covering “pre-operation, intra-operation, and post-operation.” In this context, a more holistic approach could be considered in the future, such as a multimodal intraoperative platform that combines real-time imaging (e.g., ultrasound, fluorescence) with AI-driven predictive models and AR/VR-guided navigation, which can address the dynamic complexity of pancreatic surgery by enabling seamless data fusion across the continuum of care. With advanced digital tools such as AR/VR and AI-driven platforms currently concentrated in high-income countries and high-volume surgery centers, potential inequalities in technology adoption have not yet been fully addressed.

Laga Boul-Atarass *et al* mentioned the application of AR systems in intraoperative positioning, but the current technology mostly relies on preoperative static models, which is difficult to cope with dynamic challenges such as tissue deformation and hemodynamic changes in real time. For example, in pancreatic surgery, when separating the pancreas from the superior mesenteric vein, intraoperative traction may lead to vein displacement, and traditional AR systems are based on preoperative models that may not be adjusted in real time, leading to doctors misjudging the location of blood vessels and increasing the risk of bleeding. The pancreas is located retroperitoneally, and intraoperative traction, ischemia, and other factors can lead to anatomical displacement, and errors in traditional registration techniques (such as the 5 mm deviation mentioned in the article) may affect the identification of key blood vessels. Wu *et al*^[3]^ demonstrated that the integration of intraoperative ultrasound elastography or fluorescein angiography data with real-time ultrasound positioning and a preoperative CT (computed tomography) model can improve the registration accuracy in dynamic environments using an AR navigation panel.

In conclusion, the limitations of a single technique and the challenges of dynamic registration in this study suggest that the development of pancreatic surgery techniques in the future needs to focus on multi-technique integration and dynamic adaptability. By building a cross-modal data interaction platform and developing real-time feedback algorithms, it can not only provide whole-process support for preoperative planning, intraoperative navigation and postoperative management, but also is expected to promote pancreatic surgery to move towards precision and individualized diagnosis and treatment. We sincerely thank the authors for their contributions to this important topic and believe that emphasizing technology integration will be key to advancing the field towards more personalized and safe pancreatic interventions. We sincerely thank you for your support of the authors and editors.

## Data Availability

The data that support the findings of this study are all included in this article.

## References

[1] Laga Boul-Atarass I Cepeda Franco C Sanmartín Sierra JD Castell Monsalve J Padillo Ruiz J. Virtual 3D models, augmented reality systems and virtual laparoscopic simulations in complicated pancreatic surgeries: state of art, future perspectives, and challenges. Int J Surg 2025;111:2613–23. 39869381 10.1097/JS9.0000000000002231 PMC12372726

[2] Schlanger D Graur F Popa C Moiș E Al Hajjar N. The role of artificial intelligence in pancreatic surgery: a systematic review. Updates Surg 2022;74:417–29. 35237939 10.1007/s13304-022-01255-z

[3] Wu X Wang D Xiang N. Augmented reality-assisted navigation system contributes to better intraoperative and short-time outcomes of laparoscopic pancreaticoduodenectomy: a retrospective cohort study. Int J Surg 2023;109:2598–607. 37338535 10.1097/JS9.0000000000000536 PMC10498855

